# Metazoan endoparasites of *Acestrorhynchus lacustris* (Actinopterygii: Acestrorhynchidae) from lagoons bordering the upper and middle São Francisco river basin, Brazil

**DOI:** 10.1590/S1984-29612022023

**Published:** 2022-04-20

**Authors:** Rayane Duarte, Michelle Daniele dos Santos-Clapp, Marilia de Carvalho Brasil-Sato

**Affiliations:** 1 Programa de Pós-graduação em Ciências Veterinárias, Instituto de Veterinária, Universidade Federal Rural do Rio de Janeiro – UFRRJ, Seropédica, RJ, Brasil; 2 Laboratório de Biologia e Ecologia de Parasitos, Departamento de Biologia Animal, Instituto de Ciências Biológicas e da Saúde, Universidade Federal Rural do Rio de Janeiro – UFRRJ, Seropédica, RJ, Brasil

**Keywords:** Anisakidae, Capillariidae, Digenea, Gnathostomatidae, Lagoon environment parasitology, Proteocephalidae, Anisakidae, Capillariidae, Digenea, Gnathostomatidae, parasitologia de ambiente lagunar, Proteocephalidae

## Abstract

The endoparasitic fauna of *Acestrorhynchus lacustris* from eight marginal lagoons of the upper and middle São Francisco river basin, Brazil, is recorded here for the first time. For this, a total of 106 specimens of *A. lacustris* were collected. Eighteen helminth species were found. The taxa recorded were phylum Platyhelminthes: one metacercaria of *Clinostomum* sp. (Trematoda: Clinostomidae) and plerocercoid larvae of unidentified species (Eucestoda: Proteocephalidae gen. sp.); phylum Acanthocephala: juvenile of *Quadrigyrus* sp.; and phylum Nematoda: larvae of *Brevimulticaecum* sp., *Contracaecum* sp. Type1_,_ *Contracaecum* sp. Type2, *Hysterothylacium* sp., *Gnathostoma* sp., *Spiroxys* sp., juvenile and adult specimens of *Freitascapillaria* sp., *Paracapillaria piscicola*, Capillariidae gen. sp., *Procamallanus* (*Spirocamallanus*) *hilarii*, *Procamallanus* (*S*.) *inopinatus*, *Procamallanus* (*S*.) *saofranciscencis*, *Travassosnema travassosi paranaensis*, *Cystidicoloides fischeri* and *Spinitectus rodolphiheringi*. Proteocephalidae gen. sp., *Contracaecum* sp. Type1 and *Travassosnema t*. *paranaensis* were present in all eight lagoons with high parasitic indexes. Proteocephalidae gen. sp., *Brevimulticaecum* sp., *Gnathostoma* sp., *Freitascapillaria* sp., *P. piscicola*, Capillariidae gen. sp., *Procamallanus* (*S*.) *hilarii*, *C*. *fischeri* and *S*. *rodolphiheringi* are new records for *A. lacustris*. The known geographical distribution of *Gnathostoma* sp., *Freitascapillaria* sp., *P*. *piscicola*, Capillariidae gen. sp., *Procamallanus* (*S*.) *hilarii* and *Travassosnema t. paranaensis* has now been extended to the São Francisco river basin.

## Resumo

A fauna endoparasitária de *Acestrorhynchus lacustris* de oito lagoas marginais da bacia do alto e médio rio São Francisco, Brasil, foi registrada pela primeira vez. Para isso, um total de 106 espécimes de *A. lacustris* foi coletado. Dezoito espécies de helmintos foram encontradas. Os táxons relacionados foram do Filo Platyhelminthes: uma metacercária de *Clinostomum* sp. (Trematoda: Clinostomidae); larvas plerocercoides de espécie não identificada (Eucestoda: Proteocephalidae gen. sp.); Filo Acanthocephala: juvenil de *Quadrigyrus* sp.; e Filo Nematoda: larvas de *Brevimulticaecum* sp., *Contracaecum* sp. Tipo1_,_ *Contracaecum* sp. Tipo2, *Hysterothylacium* sp., *Gnathostoma* sp., *Spiroxys* sp.; juvenis e adultos de *Freitascapillaria* sp., *Paracapillaria piscicola*, Capillariidae gen. sp., *Procamallanus* (*Spirocamallanus*) *hilarii*, *Procamallanus* (*S*.) *inopinatus*, *Procamallanus* (*S*.) *saofranciscencis*, *Travassosnema travassosi paranaensis*, *Cystidicoloides fischeri* e *Spinitectus rodolphiheringi*. Proteocephalidae gen. sp., *Contracaecum* sp. Tipo1 e *Travassosnema t*. *paranaensis* foram compartilhadas entre todas as oito lagoas com altos índices parasitários. Proteocephalidae gen. sp., *Brevimulticaecum* sp., *Gnathostoma* sp., *Freitascapillaria* sp., *P. piscicola*, Capillariidae gen. sp., *Procamallanus* (*S*.) *hilarii*, *C*. *fischeri* e *S*. *rodolphiheringi* são novos registros para *A. lacustris*. *Gnathostoma* sp., *Freitascapillaria* sp., *P*. *piscicola*, Capillariidae gen. sp., *Procamallanus* (*S*.) *hilarii* e *Travassosnema t. paranaensis* têm sua distribuição geográfica estendida para a bacia do rio São Francisco.

## Introduction

The hydrographic basin of the São Francisco river is the largest in Brazil, and traditionally it is divided into four segments: upper (from the Serra da Canastra source to Pirapora, in the state of Minas Gerais), middle (from Pirapora to Remanso, in the state of Bahia, which is the longest stretch), sub-middle (from Remanso to Paulo Afonso, both in the state of Bahia) and lower (from Paulo Afonso, in the state of Bahia to its mouth between the states of Sergipe and Alagoas, with marine influence) (Planvasf, 1989). Its ichthyofauna is diverse, with socioenvironmental importance, especially for fishing (Godinho & Godinho, 2003).

The monotypic genus *Acestrorhynchus* Eigenmann and Kennedy, 1903 (Acestrorhynchidae: Acestrorhynchinae), comprises fourteen valid species of endemic South American fish. Among these, *Acestrorhynchus britskii* Menezes, 1969, endemic of São Francisco river basin cited as Least Concern (LC) in Red List of threatened species (ICMBio, 2021); and *Acestrorhynchus lacustris* (Lütken, 1875) is native to the São Francisco and upper Paraná river basins (Froese & Pauly, 2021). The fish of this subfamily live in lake, lagoon or river pool environments (Britski et al., 1988). They have an elongated and compressed body and a mouth provided with caniniform conical teeth and are therefore popularly known as “peixe-cachorro” (“dogfish”) (Britski et al., 1999).

Studies involving the eating habits of *A. lacustris* in the Tibagi river (Bennemann et al., 2000) and Itaipu reservoir (Hahn et al., 2000), both in the state of Paraná, have classified this fish species as preferentially piscivorous. In the hydrographic basin of the São Francisco river, studies by Gomes & Verani (2003) in the Três Marias reservoir (upper river), Pompeu & Godinho (2003) in marginal lagoons of the middle river, Luz et al. (2009) in Curralinho lagoon of the sub-middle river and Rocha et al. (2011) in the Sobradinho reservoir (between the middle and sub-middle stretches) considered the eating habits of *A. lacustris* and its congener *A.britskii*, to be exclusively piscivorous, and occasionally attributed the presence of items such as plant tissue, insects and shrimps in their diets to accidental ingestion.

Brasil-Sato (2003) compiled a list of fish parasites from the São Francisco river basin and recorded the nematodes *Contracaecum* sp., *Hysterothylacium* sp. (indicated as *Heterotyphlum* sp.), *Travassosnema travassosi* Costa, Moreira & Oliveira, 1991, and *Procamallanus* (*Spirocamallanus*) *saofranciscencis* (Moreira, Oliveira & Costa, 1994), as endoparasites of *A*. *lacustris*. Costa et al. (2011) added the first record of *Rhabdochona* (*Rhabdochona*) *acuminata* (Molin, 1860) in this fish species from the Três Marias reservoir, on the upper São Francisco river.

The aim of the current study was to register the metazoan endoparasites of *A. lacustris* specimens collected in eight lagoons bordering the upper and middle São Francisco river and expand the knowledge of freshwater fish parasitology in this important Brazilian river basin.

## Materials and Methods

A total of 106 specimens of *A. lacustris* from eight marginal lagoons in the São Francisco river basin were provided for parasitological analysis by the management team of the project “Revitalization of the marginal lagoons of upper-middle São Francisco river basin, Minas Gerais, Brazil”. This project was conducted in cooperation between the Brazilian Institute for the Environment and Renewable Natural Resources (Instituto Brasileiro do Meio Ambiente e dos Recursos Naturais Renováveis, IBAMA) and the Development Company for the São Francisco and Parnaíba Valleys (Companhia de Desenvolvimento dos Vales do São Francisco e do Parnaíba, CODEVASF).

**Figure 1:**
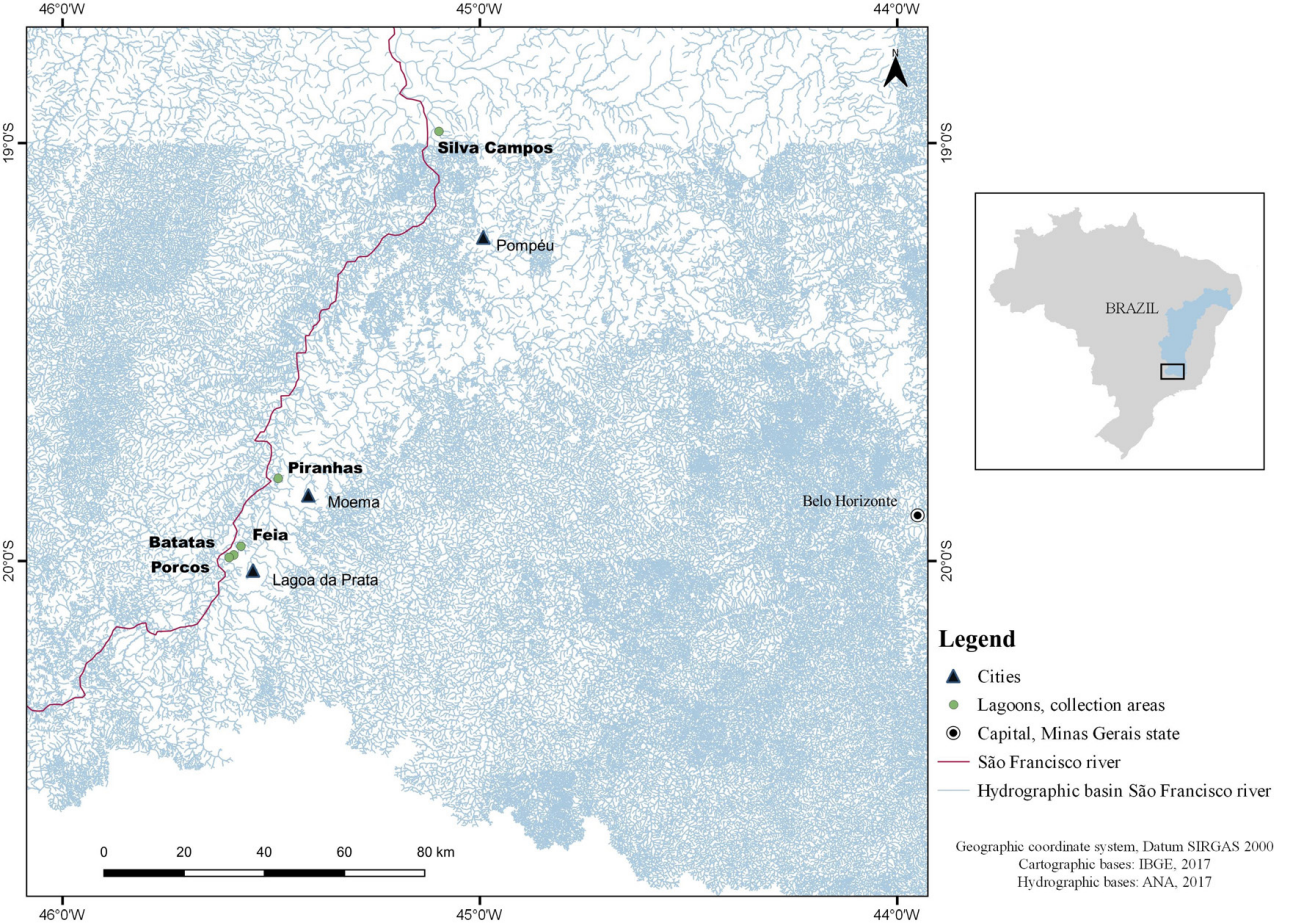
Collection areas (lagoons) for *Acestrorhynchus lacustris* in the region of the upper São Francisco river basin, state of Minas Gerais (MG), Brazil.

**Figure 2:**
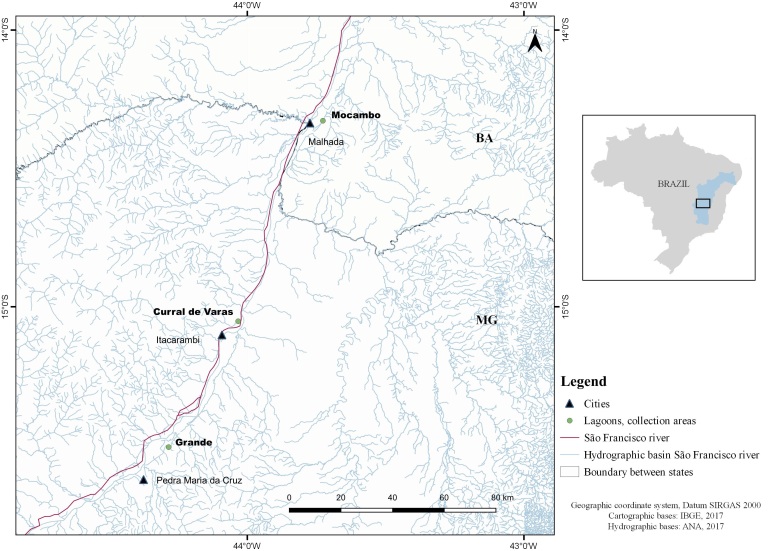
Collection areas (lagoons) for *Acestrorhynchus lacustris* in the region of the middle São Francisco river basin, states of Minas Gerais (MG) and Bahia (BA), Brazil.

Among these fish, 56 were collected from five lagoons (Batatas, Feia, Piranhas, Porcos and Silva Campos) in the upper São Francisco river basin and 50 came from three lagoons (Curral de Varas, Grande and Mocambo) in the middle São Francisco river basin (Tables 1, 2 and Figures 1-2). The software QGIS 3.14.16 with GRASS 7.8.3 was used to obtain maps (Figures 1-2). The fish were fixed in 3% formalin, marked with biometric data and the lagoon name (on tags) and individually packaged inside plastic bags. They were then sent to the Parasite Ecology and Biology Laboratory (Laboratório de Biologia e Ecologia de Parasitos, LABEPAR) of the Federal Rural University of Rio de Janeiro (Universidade Federal Rural do Rio de Janeiro, UFRRJ), Seropédica, state of Rio de Janeiro, Brazil, for examination.

**Table 1:** Localities from which *Acestrorhynchus lacustris* was collected, from lagoons in the upper and middle São Francisco river basin, states of Minas Gerais (MG) and Bahia (BA), Brazil.

**Localities (lagoons)**	**Coordinates**	**Municipality**	**Riverside bank**
**Upper**			
Porcos	19º59’31” S - 45º36’04” O	Lagoa da Prata/MG	Right
Batatas	19º59’09” S - 45º35’24” O	Lagoa da Prata/MG	Right
Feia	19º57’54” S - 45º34’22” O	Lagoa da Prata/MG	Right
Piranhas	19º48’09” S - 45º29’01” O	Moema/MG	Right
Silva Campos	18º58’18” S - 45º05’54” O	Pompéu/MG	Right
**Middle**			
Grande	15º30’27” S - 44º17’04” O	Pedras de Maria Cruz/MG	Right
Curral de Varas	15º03’09” S - 44º02’00” O	Itacarambi/MG	Left
Mocambo	14º19’40” S - 43º43’37” O	Malhada/BA	Right

**Table 2:** Numbers and sizes of *Acestrorhynchus lacustris* collected from lagoons bordering the São Francisco river basin: total number – N; number of females – NF; number of males – NM; mean total length – MTL; and respective size ranges.

**Collection localities (lagoons)**	**Collection period**	**N**	**NF**	**NM**	**MTL**	**Range**
**Upper**						
Porcos	September/2016	6	3	3	16.3	14.0 – 19.0
Batatas	September/2018	27	6	21	18.0	15.5 – 23.0
Feia	June/2011	7	6	1	24.8	23.0 – 29.0
Piranhas	June/2011	13	11	2	17.7	15.0 – 20.0
Silva Campos	October/2016	3	3	-	25.3	22.0 – 28.0
**Middle**						
					
Grande	November/2008	12	7	5	20.4	16.0 – 26.0
Curral de Varas	October/2007	15	10	5	19.7	15.5 – 25.0
Mocambo	October/2007	23	13	10	20.8	15.0 – 26.0

The procedure for fixing and preparing temporary or permanent slides of parasite specimens followed standardized methodology (Amato et al., 1991). To identify and classify the taxa, the following references were consulted: Kanev et al. (2002) for digenean metacercariae; Chervy (2002) and Chambrier et al. (2017) for eucestode plerocercoids; Amin (1987) for juvenile specimens of Acanthocephala; and Moravec (1998) for larvae, juveniles and adults of Nematoda.

Voucher specimens of parasites of *A. lacustris* were deposited in the Helminthological Collection of the Oswaldo Cruz Institute (Coleção Helmintológica do Instituto Oswaldo Cruz, CHIOC), state of Rio de Janeiro, RJ, Brazil, in accordance with the numbering presented in the results section (Table 3). The fish voucher specimen was deposited in the Zoological Museum of the University of São Paulo (Museu de Zoologia da Universidade de São Paulo, MZUSP), State of São Paulo, SP, Brazil, under the number 105.886.

**Table 3:** Occurrence of endoparasite species, deposition numbers of voucher specimens in CHIOC (Coleção Helmintológica do Instituto Oswaldo Cruz), their parasite indexes (prevalence - P; mean intensity - MI; mean abundance - MA; standard deviation - SD) and infection sites (abdominal cavity - AC; stomach - S; intestine - I; intestinal cecum - IC; liver - L), in *Acestrorhynchus lacustris* from lagoons bordering the upper and middle São Francisco river, states of Minas Gerais (MG) and Bahia (BA), Brazil.

**Endoparasites species**		**CHIOC**	**Indexes**			**Site**	**Localities (lagoons)**	
			**P (%)**	**MI ± SD**	**MA ± SD**			
**Platyhelminthes (Larvae)**								
	**Clinostomidae**							
	*Clinostomum* sp. (metacercaria)	39106	8.00	1.00	0.08 ± 0.29	AC	Grande	Middle
	**Proteocephalidae**							
	Proteocephalidae gen. sp. (plerocercoids)	.	100	101.70 ± 75.48	101.70 ± 75.48	AC; IC	Porcos	Upper
		.	22.00	20.83 ± 12.86	4.63 ± 10.09	AC; IC	Batatas	Upper
		.	100	224.30 ± 149.87	224.30 ± 149.87	AC; IC	Feia	Upper
		39123	77.0	420.00 ± 221.16	323.10 ± 265.72	AC; IC	Piranhas	Upper
		.	33.00	10.00	3.33 ± 5.77	AC; IC	Silva Campos	Upper
		.	100	281.70 ± 137.70	281.70 ± 137.70	AC; IC	Grande	Middle
		39122	100	858.60 ± 699.94	858.60 ± 699.94	AC; IC	Curral de Varas	Middle
		39124	87.00	187.00 ± 135.03	162.61 ± 141.04	AC; IC	Mocambo	Middle
**Acanthocephala (Juveniles)**								
	**Quadrigyridae**							
	*Quadrigyrus* sp.	.	4.00	1.00	0.04 ± 0.19	AC	Batatas	Upper
**Nematoda (Larvae)**								
	**Acanthocheilidae**							
	*Brevimulticaecum* sp.	39107	4.00	1.00	0.04 ± 0.21	AC	Mocambo	Middle
	**Anisakidae**							
	*Contracaecum* sp. Type1	39150	33.00	15.50 ± 13.44	5.17 ± 10.01	AC	Porcos	Upper
		39152	85.00	5.43 ± 4.91	4.63 ± 4.95	AC	Batatas	Upper
		39151	100	14.57 ± 24.76	14.57 ± 24.76	AC	Feia	Upper
		39145	100	11.69 ± 6.13	11.69 ± 6.13	AC	Piranhas	Upper
		39146	100	8.33 ± 6.66	8.33 ± 6.66	AC	Silva Campos	Upper
		39149	67.00	1.75 ± 0.71	1.17 ± 1.03	AC	Grande	Middle
		39147	53.00	1.63 ± 1.06	0.87 ± 1.13	AC	Curral de Varas	Middle
		39148	87.00	15.00 ± 17.31	13.04 ± 16.90	AC; IC; L	Mocambo	Middle
	*Contracaecum* sp. Type2	39143	4.00	1.00	0.04 ± 0.19	AC	Batatas	Upper
		39144	14.00	1.00	0.14 ± 0.38	AC	Feia	Upper
		39141	8.00	1.00	0.08 ± 0.28	AC	Piranhas	Upper
		39140	33.00	1.00	0.33 ± 0.58	AC	Silva Campos	Upper
		30139	17.00	1.00	0.17 ± 0.39	AC	Grande	Middle
		39142	7.00	1.00	0.07 ± 0.26	AC	Curral de Varas	Middle
	*Hysterothylacium* sp.	39128	44.00	3.92 ± 4.58	1.74 ± 3.58	AC	Batatas	Upper
		.	14.00	3.00	0.43 ± 1.13	AC	Feia	Upper
		39126	33.00	1.00	0.33 ± 0.58	AC	Silva Campos	Upper
		39127	20.00	1.33 ± 0.58	0.27 ± 0.59	AC; S	Curral de Varas	Middle
		39125	9.00%	2.50 ± 2.12	0.22 ± 0.85	AC	Mocambo	Middle
	**Gnathostomatidae**							
	*Gnathostoma* sp.	39108	7.00	1.00	0.07 ± 0.26	S	Curral de Varas	Middle
	*Spiroxys* sp.	39130	59.00	2.56 ± 2.19	1.52 ± 2.10	AC	Batatas	Upper
		39129	33.00	3.00	1.00 ± 1.73	AC	Silva Campos	Upper
		39132	7.00	1.00	0.07 ± 0.26	AC	Curral de Varas	Middle
		39131	39.00	2.56 ± 2.24	1.00 ± 1.86	AC; IC	Mocambo	Middle
**Nematoda (juvenile/adult)**								
	**Capillariidae**							
	*Freitascapillaria* sp.	39109	14.00	1.00	0.14 ± 0.38	AC	Feia	Upper
	*Paracapillaria piscicola*	39110	14.00	2.00	0.29 ± 0.76	AC	Feia	Upper
	Capillariidae gen. sp.	39111; 39112	29.00	1.00	0.29 ± 0.49	S; AC	Feia	Upper
	**Camallanidae**							
	*Procamallanus* (*Spirocamallanus*) *hilarii*	39117; 39118	26.00	2.00 ± 0.82	0.52 ± 0.98	S; I; IC	Batatas	Upper
	*Procamallanus* (*S.*) *inopinatus*	39138	4.00	1.00	0.04 ± 0.19	I	Batatas	Upper
		.	14.00	1.00	0.14 ± 0.38	I	Feia	Upper
		39133; 39134	33.00	2.00	0.67 ± 1.15	I	Silva Campos	Upper
		39137	17.00	2.00 ± 1.41	0.33 ± 0.89	IC	Grande	Middle
		39136	13.00	1.50 ± 0.71	0.20 ± 0.56	I	Curral de Varas	Middle
		39135	4.00	1.00	0.04 ± 0.21	IC	Mocambo	Middle
	*Procamallanus* (*S.*) *saofranciscencis*	39115	8.00	3.00	0.25 ± 0.87	I	Grande	Middle
		39116	22.00	2.00 ± 1.00	0.43 ± 0.95	I; IC	Mocambo	Middle
	**Guyanemidae**							
	*Travassosnema travassosi paranaensis*	39155	100	10.17 ± 6.88	10.17 ± 6.88	AC	Porcos	Upper
		39161	74.00	5.25 ± 2.84	3.89 ± 3.38	AC	Batatas	Upper
		39158a; 39158b	100	9.86 ± 11.99	9.86 ± 11.99	AC	Feia	Upper
		39156	92.00	11.33 ± 7.08	10.46 ± 7.47	AC	Piranhas	Upper
		39153	100	3.33 ± 1.53	3.33 ± 1.53	AC	Silva Campos	Upper
		39154a; 39154b	92.00	3.82 ± 2.60	3.50 ± 2.71	AC	Grande	Middle
		39159; 39160	80.00	6.17 ± 6.26	4.93 ± 6.11	AC	Curral de Varas	Middle
		39157	52.00	5.17 ± 4.34	2.70 ± 4.05	AC; IC	Mocambo	Middle
	**Cystidicolidae**							
	*Cystidicoloides fischeri*	39121	7.00	7.00 ± 1.41	0.52 ± 1.89	AC	Batatas	Upper
		39120	29.00	3.00 ± 1.41	0.86 ± 1.57	AC; IC	Feia	Upper
		39119	9.00	1.00	0.09 ± 0.29	IC; AC	Mocambo	Middle
	*Spinitectus rodolphiheringi*	39113; 39114	67.00	2.50 ± 0.71	1.67 ± 1.53	AC; S	Silva Campos	Upper

The ecological descriptors used were in accordance with Bush et al. (1997). Statistical tests were only applied to the parasite species that showed parasite prevalence higher than 10% (Bush et al., 1990). The software GraphPad Prism 9.2.0 was used to calculate these indexes. The frequency of dominance, the shared frequency of dominance and the mean relative dominance of each parasite species were calculated as described by Rohde et al. (1995). The ratio between the mean parasite variance and abundance (dispersion index, DI) was calculated for each parasite species to determinate its distribution pattern. The significance of the distribution was tested using the statistical *d*-test (*d* > 1.96) (Ludwig & Reynolds, 1988).

Student’s *t* test was used to check for possible differences in the total length of hosts, in relation to their sex. Pearson’s correlation coefficient (*r*) was used to assess the correlation between parasite prevalence and the hosts’ size classes, as estimated using Sturges’s formula (Sturges, 1926). Spearman’s correlation coefficient by ranks (*rs*) was used to evaluate possible correlations between the host’s total length and abundance. The chi-square test with Yate’s correction (*x*^2^ Yates) and Fisher's exact test (*F*(*p*)) were used to determine the influence of sex on the prevalence of parasites. The Mann-Whitney test (*U*) was used to assess possible differences in abundance, in relation to the hosts’ sex. These tests were applied only to species from two helminthic communities (Batatas and Mocambo lagoons), from which more than twenty fish specimens were examined. The statistical significance level adopted was *p* < 0.05 (Zar, 1996).

## Results

In total, eighteen species of helminthic endoparasites in parasite communities of *A. lacustris* from these eight marginal lagoons were identified. Two taxa in the phylum Platyhelminthes were identified: one metacercaria of *Clinostomum* sp. (Trematoda: Clinostomidae) and plerocercoid larvae of Eucestoda (Proteocephalidae gen. sp.); one taxon of the phylum Acanthocephala: Quadrigyridae - *Quadrigyrus* sp.; and fifteen taxa of the phylum Nematoda: Acanthocheilidae - *Brevimulticaecum* sp.; Anisakidae – *Contracaecum* sp. Type1 larvae of Moravec, Kohn & Fernandes, 1993, *Contracaecum* sp. Type2 larvae of Moravec, Kohn & Fernandes, 1993, and *Hysterothylacium* sp.; Gnathostomatidae – *Gnathostoma* sp., and *Spiroxys* sp.; Capillariidae – *Freitascapillaria* sp., *Paracapillaria piscicola* (Travassos, Artigas & Pereira, 1928) and unidentified species of Capillariidae gen. sp.; Camallanidae – *Procamallanus* (*Spirocamallanus*) *hilarii* Vaz & Pereira, 1934, *Procamallanus* (*Spirocamallanus*) *inopinatus* Travassos, Artigas & Pereira, 1928, and *Procamallanus* (*S.*) *saofranciscencis*; Guyanemidae – *Travassosnema travassosi paranaensis* Moravec, Kohn & Fernandes, 1993; and Cystidicolidae – *Cystidicoloides fischeri* (Travassos, Artigas & Pereira, 1928) and *Spinitectus rodolphiheringi* Vaz & Pereira, 1934.

Fourteen species of helminths were found in five lagoons in the upper São Francisco river basin and twelve species in three lagoons in the middle São Francisco river basin. The parasite indexes, sites and stages of parasite species development recorded in *A. lacustris* per lagoon and its location in the upper or middle São Francisco river basin are listed in Table 3.

Among the larval endoparasites identified (Table 3), Proteocephalidae gen. sp. and *Contracaecum* sp. Type1 occurred in eight lagoons with high parasite indexes (prevalence reaching 100% in four and three communities, respectively) with high abundance values, compared with the other species of community parasites found. These two species were followed by *Contracaecum* sp. Type2, *Hysterothylacium* sp. and *Spiroxys* sp., which occurred in six, five and four lagoons, respectively, with prevalence usually higher than 10%. *Clinostomum* sp., *Quadrigyrus* sp. *Brevimulticaecum* sp. and *Gnathostoma* sp. were found parasitizing a single specimen of *A. lacustris* from the Grande, Mocambo and Curral de Varas lagoons (which are all in the middle São Francisco river basin), respectively, with prevalence below 10% and average abundance below 1.0.

Among the juvenile and adult endohelminths represented by nematodes (Table 3), *Travassosnema t. paranaensis* stands out. This occurred in eight lagoons, in which the minimum prevalence registered was greater than 50% (reaching 100% of the fish in three lagoons in the upper São Francisco river basin), with a minimum abundance of 2.7 specimens per infected fish. This species was followed by *Procamallanus* (*S*.) *inopinatus*, which was recorded in six lagoons, with prevalence greater than 10% in four of them. *Cystidicoloides fischeri* occurred in the communities of three lagoons: two in the upper and one in the middle São Francisco river basin; and *Procamallanus* (*S*.) *saofranciscencis* occurred in two lagoons in the middle São Francisco river basin. For each of these, prevalence above 10% was found in a single lagoon, in the upper and middle São Francisco river basin, respectively.

Among these eighteen species that were components of the parasite communities of “peixes-cachorros” from São Francisco river lagoons, six (*Quadrigyrus* sp., *Freitascapillaria* sp., *P*. *piscicola*, Capillariidae gen. sp., *Procamallanus* (*S*.) *hilarii* and *S*. *rodolphiheringi*) and three (*Clinostomum* sp., *Brevimulticaecum* sp., and *Gnathostoma* sp.) helminth species were exclusive to the upper and middle São Francisco river basin, respectively. The other nine species occurred in lagoons in both stretches of the basin.

Seven species were shared between two helminth communities in the largest sampling of *A. lacustris* from the upper (Batatas lagoon) and middle (Mocambo lagoon) São Francisco river basin: four species with prevalence above 10% (Proteocephalidae gen. sp., *Contracaecum* sp. Type1, *Spiroxys* sp. and *Travassosnema t*. *paranaensis*); one species, *Hysterothylacium* sp., with prevalence below 10% in Mocambo lagoon; and two species, *Procamallanus* (*S*.) *inopinatus* and *C*. *fischeri*, with prevalence below 10% in both lagoons (Table 3).

The most dominant species in the upper São Francisco river basin were Proteocephalidae gen. sp. in the Piranhas (dominance frequency value = 10), Feia (7) and Porcos (6) lagoons; *Travassosnema t. paranaensis* in Batatas lagoon (9); and *Contracaecum* sp. Type1 in Silva Campos lagoon (2). Proteocephalidae gen. sp. was also the most dominant taxon in the three lagoons in the middle São Francisco river basin, with dominance frequency values of 20 in Mocambo, 15 in Curral de Varas and 12 in Grande. All species that had a significant statistical *d*-test result showed aggregated distribution (Table 4).

**Table 4:** Dominance frequency, shared dominance frequency, average relative dominance (standard deviation - SD), dispersion index with distribution pattern (< 1.00 - aggregated; > 1.00 - uniform; = 1.00 - random) and statistical *d*-test, regarding *Acestrorhynchus lacustris* collected from marginal lagoons in the upper and middle São Francisco river basin, states of Minas Gerais (MG) and Bahia (BA), Brazil.

**Endoparasites species**		**Dominance Frequency**	**Shared Dominance Frequency**	**Average Relative Dominance ± SD**	**Dispersion Index**			**Localities (lagoons)**	
					**Value**	**Distribution**	**Statistical d-test**		
**Platyhelminthes (Larvae)**									
	**Proteocephalidae**								
	Proteocephalidae gen. sp. (plerocercoids)	6	0	0.878 ± 0.063	56.03	Aggregated	20.67*	Porcos	Upper
		5	0	0.134 ± 0.270	21.98	Aggregated	26.67*	Batatas	Upper
		7	0	0.850 ± 0.146	100.15	Aggregated	31.35*	Feia	Upper
		10	0	0.715 ± 0.411	218.54	Aggregated	99.46*	Piranhas	Upper
		0	0	0.104 ± 0.180	10.00	Aggregated	4.59*	Silva Campos	Upper
		12	0	0.976 ± 0.018	67.32	Aggregated	33.90*	Grande	Middle
		15	0	0.985 ± 0.014	570.59	Aggregated	121.20*	Curral de Varas	Middle
		20	0	0.789 ± 0.322	122.34	Aggregated	66.81*	Mocambo	Middle
**Nematoda (Larvae)**									
	**Anisakidae**								
	*Contracaecum* sp. Type1	0	0	0.027 ± 0.042	19.39	Aggregated	10.92*	Porcos	Upper
		7	2	0.338 ± 0.313	5.30	Aggregated	9.46*	Batatas	Upper
		0	0	0.044 ± 0.047	42.07	Aggregated	19.15*	Feia	Upper
		0	0	0.089 ± 0.143	3.21	Aggregated	3.99*	Piranhas	Upper
		2	0	0.408 ± 0.096	5.32	Aggregated	2.88*	Silva Campos	Upper
		0	0	0.007 ± 0.009	0.91	Uniform	0.11	Grande	Middle
		0	0	0.002 ± 0.003	1.46	Aggregated	1.20	Curral de Varas	Middle
		2	0	0.138 ± 0262	21.90	Aggregated	24.48*	Mocambo	Middle
	*Contracaecum* sp. Type2	0	0	0.000 ± 0.001	1.00	Random	0.15	Feia	Upper
		0	0	0.010 ± 0.018	1.00	Random	0.27	Silva Campos	Upper
		0	0	0.000 ± 0.001	0.91	Uniform	0.11	Grande	Middle
	*Hysterothylacium* sp.	2	0	0.044 ± 0.077	7.36	Aggregated	12.42*	Batatas	Upper
		0	0	0.001 ± 0.003	3.00	Aggregated	2.68*	Feia	Upper
		0	0	0.026 ± 0.44	1.00	Random	0.27	Silva Campos	Upper
		0	0	0.001 ± 0.001	1.32	Aggregated	0.89	Curral de Varas	Middle
	**Gnathostomatidae**								
	*Spiroxys* sp.	2	0	0.121 ± 0.201	2.91	Aggregated	5.15*	Batatas	Upper
		0	0	0.031 ± 0.054	3.00	Aggregated	1.73	Silva Campos	Upper
		0	0	0.006 ± 0.011	3.45	Aggregated	5.77*	Mocambo	Middle
**Nematoda (juvenile/adult)**									
	**Capillariidae**								
	*Freitascapillaria* sp.	0	0	0.002 ± 0.004	1.00	Random	0.15	Feia	Upper
	*Paracapillaria piscicola*	0	0	0.002 ± 0.004	2.00	Aggregated	1.58	Feia	Upper
	Capillariidae gen. sp.	0	0	0.001 ± 0.002	0.83	Uniform	0.15	Feia	Upper
	**Camallanidae**								
	*Procamallanus* (*Spirocamallanus*) *hilarii*	0	0	0.030 ± 0.076	1.84	Aggregated	2.63*	Batatas	Upper
	*Procamallanus* (*S*.) *inopinatus*	0	0	0.001 ± 0.002	1.00	Random	0.15	Feia	Upper
		0	0	0.056 ± 0.096	2.00	Aggregated	1.10	Silva Campos	Upper
		0	0	0.002 ± 0.004	2.36	Aggregated	2.63*	Grande	Middle
		0	0	0.001 ± 0.002	1.57	Aggregated	1.44	Curral de Varas	Middle
	*Procamallanus* (*S*.) *saofranciscencis*	0	0	0.004 ± 0.012	2.05	Aggregated	2.95*	Mocambo	Middle
	**Guyanemidae**								
	*Travassosnema travassosi paranaensis*	0	0	0.095 ± 0.036	4.66	Aggregated	3.83*	Porcos	Upper
		9	2	0.326 ± 0.277	2.93	Aggregated	5.21*	Batatas	Upper
		0	0	0.097 ± 0.146	14.59	Aggregated	9.91*	Feia	Upper
		3	0	0.119 ± 0.218	5.33	Aggregated	6.52*	Piranhas	Upper
		1	0	0.232 ± 0.162	0.70	Uniform	0.06	Silva Campos	Upper
		0	0	0.015 ± 0.012	2.10	Aggregated	2.22*	Grande	Middle
		0	0	0.012 ± 0.012	7.57	Aggregated	9.36*	Curral de Varas	Middle
		0	0	0.020 ± 0.029	6.09	Aggregated	9.81*	Mocambo	Middle
	**Cystidicolidae**								
	*Cystidicoloides fischeri*	0	0	0.002 ± 0.004	2.89	Aggregated	2.57*	Feia	Upper
	*Spinitectus rodolphiheringi*	0	0	0.132 ± 0.119	1.40	Aggregated	0.63	Silva Campos	Upper

* significance value > 1.96

Female fish specimens were larger than males in three lagoons: Batatas (*t* = 4.385; *p* = 0.001), Grande (*t* = 2.899; *p* = 0.015) and Mocambo (*t* = 3.297; *p* = 0.003). In the other lagoons, although females were also larger than males, this difference was not significant (Porcos: *t* = 0.730; *p* = 0.498; Feia: *t* = 0.104; *p* = 0.921; Piranhas: *t* = 0.357; *p* = 0.728; Curral de Varas: *t* = 2.016; *p* = 0.063). In Silva Campos lagoon only females were collected.

*Contracaecum* Type1 was more abundant in larger fish (*rs* = 0.669; *p* = 0.001) and in female fish (*U* = 19.00; *p* = 0.003) from Mocambo lagoon. *Hysterothylacium* sp. was more abundant in smaller fish (*rs* = -0.459; *p* = 0.016) from Batatas lagoon, without sex correlation. The parasite indexes recorded for the other parasite species analyzed in the current study were not influenced by the host’s total length and sex (Table 5).

**Table 5:** Analysis on parasite indices, regarding possible influence of total length (*r* = Pearson's correlation coefficient; *rs* = Spearman rank correlation coefficient) and sex (*X*^2^ = chi-square with Yates correction; F(*p*) = Fisher’s exact test; and *U* = Mann-Whitney *U* test), on *Acestrorhynchus lacustris* collected from the upper (Batatas lagoon, state of Minas Gerais, MG) and middle (Mocambo lagoon, state of Bahia, BA) São Francisco river basin, Brazil.

**Endoparasites species**		**Total length**				**Sex**					**Localities (lagoons)**	
		**Prevalence**		**Abundance**		**Prevalence**			**Abundance**			
		***r***	***p***	***rs***	***p***	***X^2^***	***P***	***F* (*p*)**	***U***	***p***		
**Platyhelminthes (Larvae)**												
	**Proteocephalidae**											
	Proteocephalidae gen. sp. (plerocercoids)	-0.473	0.343	-0.288	0.145	0.003	0.095	>0.999	52.50	0.358	Batatas	Upper
		-0.354	0.492	-0.256	0.239	0.145	0.704	>0.999	46.00	0.247	Mocambo	Middle
**Nematoda (Larvae)**												
	**Anisakidae**											
	*Contracaecum sp.* Type1	0.401	0.430	0.158	0.430	0.018	0.895	>0.999	48.50	0.410	Batatas	Upper
		0.282	0.589	0.669	0.001*	2.230	0.135	0.069	19.00	0.003*	Mocambo	Middle
	*Hysterothylacium* sp.	-0.774	0.071	-0.460	0.016*	1.181	0.277	0.182	36.50	0.105	Batatas	Upper
	**Gnathostomatidae**											
	*Spiroxys* sp.	-0.556	0.252	-0.033	0.869	0.003	0.958	0.662	59.00	0.837	Batatas	Upper
		-0.676	0.140	-0.346	0.106	0.006	0.940	>0.999	64.50	>0.999	Mocambo	Middle
**Nematoda (juvenile/adult)**												
	**Camallanidae**											
	*Procamallanus* (*Spirocamallanus*) *hilarii*	-0.545	0.296	-0.068	0.738	0.003	0.953	>0.999	53.00	0.411	Batatas	Upper
	*Procamallanus* (*S.*) *saofranciscencis*	-0.353	0.493	-0.026	0.905	0.472	0.492	0.339	51.50	0.254	Mocambo	Middle
	**Guyanemidae**											
	*Travassosnema travassosi paranaensis*	-0.056	0.916	-0.006	0.977	0.220	0.639	0.633	56.60	0.717	Batatas	Upper
		-0.071	0.894	0.328	0.127	0.365	0.546	0.414	50.00	0.337	Mocambo	Middle

* significant values (significance level *p* < 0.05).

The richness of the parasite communities of *A. lacustris* in these eight lagoons was based on a minimum of three species (Proteocephalidae gen. sp., *Contracaecum* sp. Type1 and *Travassosnema t. paranaensis*), which were shared among all communities. In the lagoons of the upper São Francisco river basin, the parasite richness of *A*. *lacustris* ranged from three to ten species of helminths. The parasite communities from the Batatas and Feia lagoons in the upper São Francisco river basin presented the greatest richness (ten species each), while Silva Campos lagoon had eight species, Piranhas had four species and Porcos had the smallest number of species recorded (three species). Among the lagoons of the middle São Francisco river basin, the highest richness occurred in the parasite community of *A*. *lacustris* from Mocambo lagoon (nine species), followed by Curral de Varas Lagoon (eight species) and Grande Lagoon (seven species).

## Discussion

The diet of *A. lacustris* in this study consisted mainly of fish, thus reinforcing the previous reports regarding the piscivorous habit of acestrorhynchid fish in the São Francisco river basin (Gomes & Verani, 2003; Pompeu & Godinho, 2003; Luz et al., 2009; Rocha et al., 2011). Bell & Burt (1991) mentioned that piscivorous fish shelter more endoparasite species than do non-piscivorous fish, because their diet includes smaller species of fish (foragers), which had previously become infected. Smaller fish acted as intermediate or paratenic hosts for parasite groups, especially nematodes in the upper São Francisco river basin (Costa et al., 2011; Albuquerque et al., 2016).

In the present study, as expected, *A*. *lacustris* was a piscivorous predator that acted as the definitive host of nine parasite species (*Freitascapillaria* sp., *P. piscicola*, Capillariidae gen. sp., *Procamallanus* (*S*.) *hilarii*, *Procamallanus* (*S*.) *inopinatus, Procamallanus* (*S*.) *saofranciscencis*, *Travassosnema t. paranaensis*, *C. fischeri* and *S*. *rodolphiheringi*). However, the parasite community of *A*. *lacustris* also included nine helminth species (50%) in the larval stage, some of them with high indexes (abundance and prevalence). This highlights the importance of *A*. *lacustris* as an intermediate or paratenic host too.

Piscivorous fish parasitized by helminth larvae can be preyed upon by large vertebrates (birds, mammals and reptiles) that live in or visit the lagoons. This increases the availability of the respective definitive hosts for these helminths and the opportunity that these helminths have for reaching them. However, the invasive parasitic niches (autonomously, or through intermediate or paratenic hosts) need to overlap with the hosts’ niches, for infection to occur (Rolbiecki, 2006). The occurrence of parasite species in the larval stage, proportional to adults and juveniles, is also indicative of this overlap in the lagoon environment.

Dobson & Roberts (1994) confirm earlier studies that suggest that increasing degrees of aggregation are crucial in allowing several species of parasites to coexist in the same species of hosts. In this study, the species Proteocephalidae gen. sp., *Contracaecum* sp. Type1, *Hysterothylacium* sp., *Spiroxys* sp., *Procamallanus* (*S.*) *hilarii*, *Procamallanus* (*S.*) *inopinatus*, *Procamallanus* (*S.*) *saofranciscencis*, *Travassosnema t. paranaensis* and *C. fischeri* whose statistical *d*-tests were significant, showed the typical pattern of aggregated distribution. This tends to increase the stability in the parasite-host relationship to achieve the parasites’ reproductive success, because the higher the level of aggregation of the parasites, the lower the rates of pathogenicity and mortality of the hosts induced by the parasites (Zuben, 1997).

Mature specimens of proteocephalids have been recorded in the following top-of-chain predatory fish species in the upper São Francisco river: Characiformes – *Salminus brasiliensis* (Cuvier, 1816) (= *Salminus franciscanus* Lima & Britski, 2007) (Brasil-Sato, 2003); Siluriformes – *Pseudoplatystoma corruscans* (Spix & Agassiz, 1829) (Brasil-Sato, 2003); and Perciformes – *Cichla kelberi* Kullander & Ferreira, 2006 (Santos-Clapp & Brasil-Sato, 2014). Plerocercoids have been reported in many fish species in the upper São Francisco river: Characiformes - *Prochilodus argenteus* Spix & Agassiz, 1829 (Monteiro et al., 2009), *P. piraya* (Santos-Clapp et al., 2022, in press), *Salminus hilarii* Valenciennes, 1850 (Duarte et al., 2016) and *Tetragonopterus chalceus* Spix & Agassiz, 1829 and *Triportheus guentheri* (Garman, 1890) (Albuquerque et al., 2016); and Siluriformes - *Pimelodus maculatus* Lacepède, 1803 (Brasil-Sato, 2003) and *Pimelodus pohli* Ribeiro & Lucena, 2006 (Sabas & Brasil-Sato, 2014). In *A*. *lacustris*, which is an expert predator, only the larvae of these proteocephalids formed part of their parasite community, but with elevated indexes, and these were dominant in the communities of six out of these eight lagoons sampled in the upper and middle São Francisco river.

Larval specimens of *Rhabdochona* (*R*.) *acuminata* and *Rhabdochona* sp. have been registered in the abdominal cavity of some species of fish from the upper São Francisco river (Brasil-Sato, 2003; Brasil-Sato & Santos, 2005; Costa et al., 2011; Santos-Clapp & Brasil-Sato, 2014; Albuquerque et al., 2016). This indicates that this nematode species is not host-specific at this development stage. Occurrence of adult specimens of *Rhabdochona* (*R.*) *acuminata* in *A. britskii* and *A. lacustris* from the Três Marias reservoir, in the upper São Francisco river, was registered by Costa et al. (2011) and their absence from the *A*. *lacustris* specimens from the lagoons of the present study is indicative that the richness estimated for these lagoon parasite communities may be even higher than what was found in this study. The absence of juvenile or adult nematode specimens may have been due to the small numbers of definitive hosts collected in some lagoons (i.e. Porcos, Feia and Silva Campos), or because the foraging fish in the lagoons were not infected with rhabdochonid larvae at the time of sampling these fish.

In addition to the descriptions and records in the upper São Francisco river, endohelminths parasitizing *A. lacustris* have also been found in other localities: *T. travassosi* (Nematoda) in the Tibagi river, state of Paraná (Silva-Souza & Saraiva, 2002); *Clinostomum* sp. and *Rhipidocotyle gibsoni* Kohn & Fernandes, 1994 (Digenea), *Quadrigyrus torquatus* Van Cleave, 1920 (Acanthocephala) and *Contracaecum* sp. Type1, *Contracaecum* sp. Type2, *Contracaecum* sp., *Eustrongylides* sp. and *Procamallanus* sp. (Nematoda) in marginal lagoons of the upper Paraná river (Carvalho et al., 2003); Philometridae gen. sp. (Nematoda) (Takemoto et al., 2009); *Philonema* sp. “A” of Buhrnheim, 1976, and *Procamallanus* (*S*.) *saofranciscencis* (Nematoda) (Eiras et al., 2010); and *Austrodiplostomum compactum* (Lutz, 1928) (Digenea), Cestoda fam. gen. sp. and Onchoproteocephalidea gen. sp. (Lehun et al., 2020) on the floodplain of the upper Paraná river, which is another natural basin for this host.

Silva-Junior et al. (2011) registered parasitism by Anisakidae larvae in specimens of *A. lacustris* collected in an environmental protection area of the Curiaú river, Macapá, state of Amapá, along with the representatives of this family (*Contracaecum* spp. and *Hysterothylacium* sp.) and Gnathostomatidae (*Gnathostoma* sp.) that were also collected in the “peixes-cachorros” of the present study. These deserve attention due to the potential zoonotic risk. In the Peixe river, state of São Paulo, Abdallah et al. (2012) recorded *Contracaecum* sp., *Dioctophyme renale* (Goeze, 1782), *Philometroides caudata* Moravec, Scholz & Vivas-Rodríguez, 1995, *Procamallanus* (*S.*) *inopinatus*, *Procamallanus* (*S*.) *neocaballeroi* (Caballero-Deloya, 1977) and *Procamallanus* (*S*.) *saofranciscencis*; while Camargo et al. (2015) registered *Ascocotyle* sp., Diplostomidae gen. sp. and *Sphincterodiplostomum musculosum* Dubois, 1936 (Digenea) and *Contracaecum* sp., *P. caudata*, *Procamallanus* (*S*.) *inopinatus* and *Procamallanus* (*S*.) *saofranciscencis* (Nematoda). In the Batalha river, state of São Paulo, the following were recorded: *A. compactum*, *Bellumcorpus major* Kohn, 1962, *R. santanaensis* and *Rhipidocotyle gibsoni* Kohn & Fernandes, 1994 (Digenea) and Capillariidae gen. sp., *Contracaecum* sp., *Goezia brasiliensis* Moravec, Kohn & Fernandes, 1994, *Guyanema raphiodoni* Moravec, Kohn & Fernandes, 1993, *T. travassosi*, *P. caudata, Spiroxys contortus* (Rudolphi, 1819), *Heliconema* sp. and *Procamallanus* (*S*.) *inopinatus* (Nematoda) (Pedro et al., 2016a); *Rhipidocotyle santanaensis* Lunaschi, 2004 (Digenea) (Pedro et al., 2016b); and *Contracaecum* sp. as a bioindicator of metal pollution (Leite et al., 2017).

The parasite records relating to *A. lacustris* mostly include nematode species (larval and adult specimens), followed by digeneans. In the present study, the helminthic community included elevated presence of nematodes (richness and abundance), but regarding digeneans, only one specimen of *Clinostomum* sp. was collected, from the abdominal cavity of *A. lacustris* from Grande lagoon. This result, with absence of adult specimens of Digenea and Eucestoda in the parasite community of *A. lacustris*, reflects the possibility that the food items available, which formed the prey of *A. lacustris*, did not include gastropod molluscs or aquatic invertebrates as intermediate hosts in the evolutionary cycles of these parasites. It may even reflect a possibility that infective forms of these species evolve to use definitive hosts other than “peixes-cachorros” (*e.g.,* Proteocephalidae gen. sp.).

The highest number of helminth species (ten species) was found in the parasite communities of Batatas and Feia lagoons (upper São Francisco river basin), in which the richness of the infracommunities ranged from one to six species among the twenty-seven infected fish and from three to six species among the seven infected fish, respectively. Another community that stood out was Silva Campos lagoon (upper São Francisco river basin), with eight species of parasites in only three infected fish, in which the infracommunity richness ranged from four to five parasite species per host. In two lagoons, Feia and Silva Campos, the number of fish examined was low (seven and three fish, respectively) and the fish were larger than those in the other lagoons (average total length = 24.8 and 25.3 cm, respectively). The parasite community of *A. lacustris* from Mocambo lagoon (middle São Francisco river basin) showed the highest richness of the parasite infracommunity (one to seven helminth species per fish) and presented nine helminth species.

The parasite communities of *A. lacustris* from these lagoons in the upper and middle São Francisco river are structured by constant species that are shared with high parasite indexes. *Travassosnema travassosi* is a species whose adult specimens are well correlated with acestrorhynchid hosts (Cypriniformes): in the São Francisco river, state of Minas Gerais (Costa et al., 1991); in the Tibagi river, state of Paraná (Silva-Souza & Saraiva, 2002); and in the Batalha river, state of São Paulo (Pedro et al., 2016a).

In addition, it was possible to detect rare species (*e.g*., *Brevimulticaecum* sp. and *Gnathostoma* sp.) in the communities from the lagoons along the basin, even with the sampling limitation presented in this study. Batatas and Feia (upper São Francisco river lagoons) and Mocambo (middle São Francisco river lagoon) showed the highest richness of the parasite communities of *A. lacustris*. Because of the inherent characteristics of the locations of the Feia and Mocambo lagoons, they receive less input of water from the São Francisco river basin. Thus, water inflow to them is rarer than to the other lagoons. This regional characteristic, together with the lower water input, alters the density of the populations of organisms that survive in its environment and consequently the composition of the parasite fauna and its indexes. Feia lagoon not only had the largest number of species registered (ten), but also had the largest number of adult helminth species (six). As all organisms involved in the cycles of these parasites had long periods of exposure to them in the same area, transmission of some species of parasites was favored. Interaction with and predation of smaller fish by the “peixes-cachorros” must have facilitated occurrence of the rare parasite species detected in this lagoon, such as the three capillariid nematode species found (juveniles and adults), which had never been found in fish from the upper São Francisco river. These rare species contributed to the increase in parasite richness. The presence of juvenile and adult helminth species in the communities of these lagoons also suggest that dynamism of accumulation of parasites exists, with ongoing infections and reinfections. This would be dependent on the life cycles of the parasites (in many cases unknown), the trophic mechanisms and the particularities of the lagoons, which need to be studied in greater detail.

These lagoons have been recognized for their importance as fundamental “nurseries” for reproduction of many fish species (Sato & Godinho, 2003), including the foraging fish that *A. lacustris* feeds on (Pompeu & Godinho, 2003). They are complex ecosystems for which investigation of parasitism assists in understanding the supporting biotic interactions that exist in them.

## Conclusions

This was the first study on the parasites of *A*. *lacustris* in the São Francisco river and was a pioneer regarding studies on fish parasites from lagoons in this hydrographic basin. The composition of the parasite community of these piscivorous fish, collected from eight lagoons in the upper and middle São Francisco river basin, comprised eighteen species of helminths (of seven families, including Anisakidae and Gnathostomatidae, which are important due to the zoonotic risk that they pose), from heteroxenous cycles that were established through predation of intermediate hosts by *A*. *lacustris*. There was a minimum of three shared species (Proteocephalidae gen. sp., *Contracaecum* sp. Type1 and *Travassosnema t*. *paranaensis*) between the parasite communities in these lagoons, which all had high parasite indexes. The correlation between adult *Travassosnema t*. *paranaensis* helminths and acestrorhynchids can be highlighted.

Nine parasite species were recorded for the first time in *A*. *lacustris*: Proteocephalidae gen. sp., *Brevimulticaecum* sp., *Gnathostoma* sp., *Freitascapillaria* sp., *P. piscicola*, Capillariidae gen. sp., *Procamallanus* (*S*.) *hilarii, C*. *fischeri* and *S*. *rodolphiheringi*. The geographical distribution of six nematode species: three capillariid species, *Gnathostoma* sp., and *Procamallanus* (*S*.) *hilarii* (with low parasite indexes) and *Travassosnema t. paranaensis*, was expanded to the São Francisco river basin through the present study.

The parasite community of *A. lacustris* from lagoons bordering the upper and middle São Francisco river basin reflects occurrences of many biological interactions and several parasites’ life cycles. The lagoons of this important Brazilian hydrographic basin maintain part of the biodiversity of the Cerrado biome, and efforts are needed to expand the knowledge about them. Based on the current survey, it can be estimated that the parasite richness of *A. lacustri*s from the lagoons of the upper and middle São Francisco river basin is even higher than what was recorded in this study.

## References

[B001] Abdallah VD, Azevedo RK, Carvalho ED, Silva RJ (2012). New hosts and distribution records for nematode parasites of freshwater fishes from São Paulo State, Brazil. Neotrop Helminthol.

[B002] Albuquerque MC, Santos-Clapp MD, Brasil-Sato MC (2016). Endoparasites of two species of forage fish from the Três Marias reservoir, Brazil: new host records and ecological indices. Rev Bras Med Vet.

[B003] Amato JFR, Boeger WA, Amato SB (1991). Protocolos para laboratório: coleta e processamento de parasitos do pescado.

[B004] Amin OM (1987). Key to the families and subfamilies of Acanthocephala, with the erection of a new class (Polyacanthocephala) and a new order (Polyacanthorhynchida). J Parasitol.

[B005] Bell G, Burt A (1991). The comparative biology of parasite species diversity: internal helminths of freshwater fish. J Anim Ecol.

[B006] Bennemann ST, Shibata OA, Garavello JC (2000). Peixes do rio Tibagi: uma abordagem ecológica..

[B007] Brasil-Sato MC, Godinho HP, Godinho AL (2003). Águas, peixes e pescadores do São Francisco das Minas Gerais..

[B008] Brasil-Sato MC, Santos MD (2005). Metazoan parasites of *Conorhynchos conirostris* (Valenciennes, 1840) an endemic siluriform fish of the São Francisco basin, Brazil. Rev Bras Parasitol Vet.

[B009] Britski HA, Sato Y, Rosa ABS (1988). Manual de identificação de peixes da região de Três Marias (com chaves de identificação para os peixes da Bacia do São Francisco).

[B010] Britski HA, Silimon KZS, Lopes BS (1999). Peixes do Pantanal: manual de identificação..

[B011] Bush AO, Aho JM, Kennedy CR (1990). Ecological versus phylogenetic determinants of helminth parasite community richness. Evol Ecol.

[B012] Bush AO, Lafferty KD, Lotz JM, Shostak AW (1997). Parasitology meets ecology on its own terms: Margolis et al. revisited. J Parasitol.

[B013] Camargo AA, Pedro NHO, Pelegrini LS, Azevedo RK, Silva RJ, Abdallah VD (2015). Parasites of *Acestrorhynchus lacustris* (Lütken, 1875) (Characiformes: Acestrorhynchidae) collected from the Peixe River, southeast Brazil. Acta Sci Biol Sci.

[B014] Carvalho SD, Guidelli GM, Takemoto RM, Pavanelli GC (2003). Aspectos ecológicos da fauna endoparasitária de *Acestrorhynchus lacustris* (Lütken, 1875) (Characiformes, Acestrorhynchidae) da planície de inundação do alto rio Paraná, Brasil. Acta Sci Biol Sci.

[B015] Chambrier A, Scholz T, Mariaux J, Kuchta R, Caira JN, Jensen K (2017). Planetary biodiversity inventory (2008-2017): Tapeworms from vertebrate bowels of the Earth..

[B016] Chervy L (2002). The terminology of larval cestodes or metacestodes. Syst Parasitol.

[B017] Costa DPC, Albuquerque MC, Brasil-Sato MC (2011). *Rhabdochona* (*Rhabdochona*) *acuminata* (Nematoda) em peixes (Characiformes, Acestrorhynchidae) do reservatório de Três Marias, alto rio São Francisco, Brasil. Neotrop Helminthol.

[B018] Costa HMA, Moreira NIB, Oliveira CL (1991). *Travassosnema* gen. n. with the description of *T. travassosi* sp. n. (Dracunculoidea, Guyanemidae) parasite of *Acestrorhynchus lacustris* Reinhardt, 1874 (Characidae) from Três Marias Reservoir, MG, Brazil. Mem Inst Oswaldo Cruz.

[B019] Dobson A, Roberts M (1994). The population dynamics of parasitic helminth communities. Parasitology.

[B020] Duarte R, Santos-Clapp MD, Brasil-Sato MC (2016). Endohelminthes of *Salminus hilarii* Valenciennes (Actinopterygii: Bryconidae) and their ecological descriptors in the upper São Francisco River, Brazil. Rev Bras Med Vet.

[B021] Eiras JC, Takemoto RM, Pavanelli GC (2010). Diversidade dos parasitas de peixes de água doce do Brasil..

[B022] Froese R, Pauly D (2021). Acestrorhynchus lacustris.

[B023] Godinho AL, Godinho HP (2003). Águas, peixes e pescadores do São Francisco das Minas Gerais..

[B024] Gomes JHC, Verani JR, Godinho HP, Godinho AL (2003). Águas, peixes e pescadores do São Francisco das Minas Gerais..

[B025] Hahn NS, Delariva RL, Loureiro VE (2000). Feeding of *Acestrorhynchus lacustris* (Characidae): a post impoundment studies on Itaipu reservoir, upper Paraná River, PR. Braz Arch Biol Technol.

[B026] ICMBio (2021). Acestrorhynchus britskii.

[B027] Kanev I, Radev V, Fried B, Gibson DL, Jones A, Bray RA (2002). Keys to the Trematoda..

[B028] Lehun AL, Hasuike WT, Silva JOS, Ciccheto JRM, Michelan G, Rodrigues AFC (2020). Checklist of parasites in fish from the upper Paraná River floodplain: an update. Rev Bras Parasitol Vet.

[B029] Leite LAR, Pedro NHO, Azevedo RK, Kinoshita A, Gennari RF, Watanabe S (2017). *Contracaecum* sp. parasitizing *Acestrorhynchus lacustris* as a bioindicator for metal pollution in the Batalha River, southeast Brazil. Sci Total Environ.

[B030] Ludwig JA, Reynolds JF (1988). Statistical ecology: a primer on methods and computing..

[B031] Luz SCS, El-Deir ACA, França EJ, Severi W (2009). Estrutura da assembléia de peixes de uma lagoa marginal desconectada do rio, no submédio Rio São Francisco, Pernambuco. Biota Neotrop.

[B032] Monteiro CM, Santos MD, Zuchi NA, Brasil-Sato MC (2009). Ecological parameters of the endohelminths in relation to size and sex of *Prochilodus argenteus* (Actinopterygii: Prochilodontidae) from the Upper São Francisco River, Minas Gerais, Brazil. Zoologia.

[B033] Moravec F (1998). Nematodes of Freshwater Fishes of the Neotropical Region..

[B034] Pedro NHO, Pellegrini LS, de Azevedo RK, Abdallah VD (2016). Biodiversity of metazoan parasites in *Acestrorhynchus lacustris* (Lütken, 1875) (Characiformes: Acestrorhynchidae) from the Batalha River, São Paulo State, Brazil. Pan Am J Aquatic Scien.

[B035] Pedro NHO, Pelegrini LS, Azevedo RK, Abdallah VD (2016). First record of *Rhipidocotyle santanaensis* (Digenea) parasitizing *Acestrorhynchus lacustris* from Batalha River, Brazil. Braz J Biol.

[B036] PLANVASF (1989). Plano diretor para o desenvolvimento do vale do São Francisco – Relatório final..

[B037] Pompeu PS, Godinho HP, Godinho HP, Godinho AL (2003). Águas, peixes e pescadores do São Francisco das Minas Gerais..

[B038] Rocha AAF, Santos NCL, Pinto GA, Medeiros TN, Severi W (2011). Diet composition and food overlap of *Acestrorhynchus britskii* and *A. lacustris* (Characiformes: Acestrorhynchidae) from Sobradinho reservoir, São Francisco River, Bahia State. Acta Sci Biol Sci.

[B039] Rohde K, Hayward C, Heap M (1995). Aspects of the ecology of metazoan ectoparasites of marine fishes. Int J Parasitol.

[B040] Rolbiecki L (2006). Correlation between the occurrence of parasites and body length of roach, carp bream, European perch, zander, and ruffe in the Vistula Lagoon estuary. Oceanol Hydrobiol Stud.

[B041] Sabas CSS, Brasil-Sato MC (2014). Helminth fauna parasitizing *Pimelodus pohli* (Actinopterygii: Pimelodidae) from the upper São Francisco River, Brazil. Rev Bras Parasitol Vet.

[B042] Santos-Clapp MD, Brasil-Sato MC (2014). Parasite Community of *Cichla kelberi* (Perciformes, Cichlidae) in the Três Marias Reservoir, Minas Gerais, Brazil. Rev Bras Parasitol Vet.

[B043] Santos-Clapp MD, Duarte R, Albuquerque MC, Brasil-Sato MC (2022). Helminth endoparasites of endemic fish *Pygocentrus piraya* (Characiformes, Serrasalmidae) from Três Marias reservoir, Minas Gerais, Brazil. An Acad Bras Cienc.

[B044] Sato Y, Godinho HP, Carolsfeld J, Harvey B, Ross C, Baer A (2003). Migratory fishes of South America: Biology, Fisheries, and Conservation Status..

[B045] Silva-Junior ACS, Ramos JS, Gama CS (2011). Parasitismo de larvas de Anisakidae em *Acestrorhynchus lacustris* da área de proteção ambiental do rio Curiaú, Macapá, estado do Amapá. Rev Bras Eng Pesca.

[B046] Silva-Souza AT, Saraiva A (2002). Ecological data of *Tavassosnema travassosi travassosi* (Dracunculoidea: Guyanemidae) from humour of the eyes of *Acestrorhynchus lacustris* from Tibagi River, Paraná, Brazil. Mem Inst Oswaldo Cruz.

[B047] Sturges HA (1926). The choice of a class interval. J Am Stat Assoc.

[B048] Takemoto RM, Pavanelli GC, Lizama MAP, Lacerda ACF, Yamada FH, Moreira LHA (2009). Diversity of parasites of fish from the Upper Paraná River floodplain, Brazil. Braz J Biol.

[B049] Zar JH (1996). Biostatistical analysis..

[B050] Zuben CJV (1997). Implicações da agregação espacial de parasitas para a dinâmica populacional na interação hospedeiro-parasita. Rev Saude Publica.

